# Implementation of a 3D ocean model to understand upland lake wind-driven circulation

**DOI:** 10.1007/s10652-017-9548-6

**Published:** 2017-10-12

**Authors:** L. A. Morales-Marín, J. R. French, H. Burningham

**Affiliations:** 1 UCL Department of Geography, Environmental Change Research Centre, University College London, Gower Street, London, UK WC1E 6BT UK; 2 Present Address: Global Institute for Water Security, University of Saskatchewan, 11 Innovation Boulevard, Saskatoon, SK S7N 3H5 Canada

**Keywords:** Hydrodynamics, 3D model, Small upland lake, Wind-driven circulation

## Abstract

A community numerical ocean model is used to extend the understanding of wind-driven circulation in small upland lakes. A 3D model of a case study lake (Llyn Conwy, Wales, UK) is calibrated against measured velocity profiles via adjustment of the bottom roughness coefficient. Validation against a separate set of measured velocity profiles confirms the ability of the model to resolve key features of the flow field. Sensitivity analysis shows that the velocity field responds rapidly to changes in the wind forcing. Analysis of the gross circulation using Empirical Orthogonal Functions reveals a persistent two-gyre circulation pattern in the upper half layer of the water column driven by the interaction of wind and bathymetry. At the bottom, the flow is characterised by locally strong currents and analysis of vertical circulation over short time scales shows strong currents in the deepest parts of the lake basin and the responsiveness of the water column to changes in wind speed and direction. Even in small lakes, the assumption of uniform wind stress across the water surface is not always justified and topographic sheltering or other catchment roughness effects give rise to heterogeneity in the wind field. An idealized experiment for the case study lake shows that differences in circulation emerge if the wind stress is allowed to vary across the lake. Energetic wind forcing in upland areas can drive an energetic lake circulation that has important implications for mixing and sediment dynamics. 3D numerical modelling of wind-driven circulation should be more widely used to provide insights into physical limnology to support a wide range of ecological, biogeochemical and palaeoenvironmental studies.

## Introduction

Studies of the occurrence and size distribution of surface freshwater bodies have highlighted the importance of small lakes, especially those smaller than 1 km$^2$ (e.g. [16, 45]). Small lakes have disproportionately high hydrological and nutrient processing rates [62], greater diel temperature ranges [69] and their sheer number means that they are a significant contributor to global geochemical cycles and elemental budgets [24]. Despite their importance, many aspects of their physical limnology are still not adequately understood. This is especially true of small upland lakes, which can have complex hydrodynamic and thermal regimes on account of more energetic wind forcing than their lowland counterparts. Upland lakes have been extensively studied, not least their sedimentary records, which provide valuable archives of both catchment and wider environmental change [3, 28]. However, the nature of their wind-driven circulation remains under-researched and this constitutes a knowledge gap that has implications for palaeoenvironmental as well as hydrological and biogeochemical studies.

From a physical perspective, lakes are dominated by three sets of external forcings: heat flux exchanges and thermal forcings, inflows and outflows, and wind. Wind forcing is a key factor determining the water circulation and also provides an energy source for vertical mixing. Wind energy is converted into turbulence in the surface layer and is then transferred to the lower parts of the epilimnion by turbulent diffusion, until the thermal gradient dissipates the energy [63]. Turbulent mixing in a lake has a layered vertical structure, because the water motion is largely confined to the epilimnion and currents in the hypolimnion are weak [2, 15]. In shallow lakes, wind-induced turbulence may occur at all depths, and therefore can significantly enhance nutrient entrainment from the sediment bed as well as intermittently re-suspending bottom sediments [28, 32].

Given the complexities of lake hydrodynamics, numerical modelling is increasingly popular as a means of characterising the circulation, mixing and stratification processes that control the transport and deposition of sediments [8, 9, 38, 54]. For studies of vertical thermal structure and density stratification, especially in small lakes, one-dimensional (1D) models have been used with some predictive success, and also coupled with biogeochemical and ecosystem models [5, 17, 26, 57, 66]. Two-dimensional (2D) (vertically averaged) schemes have been used in a few cases where depth-variation in velocity or temperature is not significant, although this is usually the case only in very shallow water bodies [20]. In most lakes, three-dimensional (3D) schemes are needed. These usually assume a hydrostatic pressure distribution (e.g. [33, 64]).

Since the 1990s, 3D schemes have improved greatly in their capability and the enormous developments in computing mean that models that would once have required a super-computer can now be run on the desktop. The wide variety of 3D model codes includes commercially-developed packages such as *DELFT3D* [41] as well as open-source community models (e.g. *POM*, [6]; *FVCOM*, [10]). Although all essentially solve the same 3D Navier–Stokes equations, they differ in terms of their approach to turbulence closure, numerical solution, discretization in space and treatment of boundary conditions. 3D models still incorporate empirical coefficients that must be calibrated by reference to observations, and remain highly dependent upon the quality of the data used to calibrate and force them [7, 32].

In limnology, 3D models have been most commonly used for large lakes [13, 31, 40]. In small lakes, 3D hydrodynamic modelling has been performed to investigate the fate and transport of buoyant storm-river water and the implications of plume mixing dynamics on lake ecological functioning [56]. Other recent studies have investigated the effects of ice layers on small lake hydrodynamics and thermal structure [50], and the effect of wind-driven circulation on phytoplankton distribution [68]. However, the 3D modelling of small upland lakes remains uncommon, probably because 1D models are considered adequate to resolve the evolution of lake thermal structure at seasonal and longer timescales [19]. Studies in which sediment resuspension has been of interest have usually resorted to empirical formulations [23]. Even in small lakes, however, 3D models offer clear advantages in their ability to resolve the complexity of the interaction between sediments and the water column, especially where a strong wind-driven circulation is present.

This work presented here aims to advance the understanding of wind-driven circulation in small upland lakes through 3D modelling. The work is motivated by the realisation that energetic wind forcing can generate complex current patterns that have potentially important implications for a variety of ecological and palaeoenvironmental studies and which cannot be resolved using 1D models. Specifically, a numerical hydrodynamic ocean model is calibrated and validated against field measurements of flow velocity profiles and circulation pattern for a case study lake (Llyn Conwy, North Wales, UK). The model is used to investigate the sensitivity of lake circulation to meteorological forcing, including the effects of spatial heterogeneity of wind forcing on horizontal circulation patterns.

## Methods

### 3D hydrodynamic model description

**Fig. 1:**
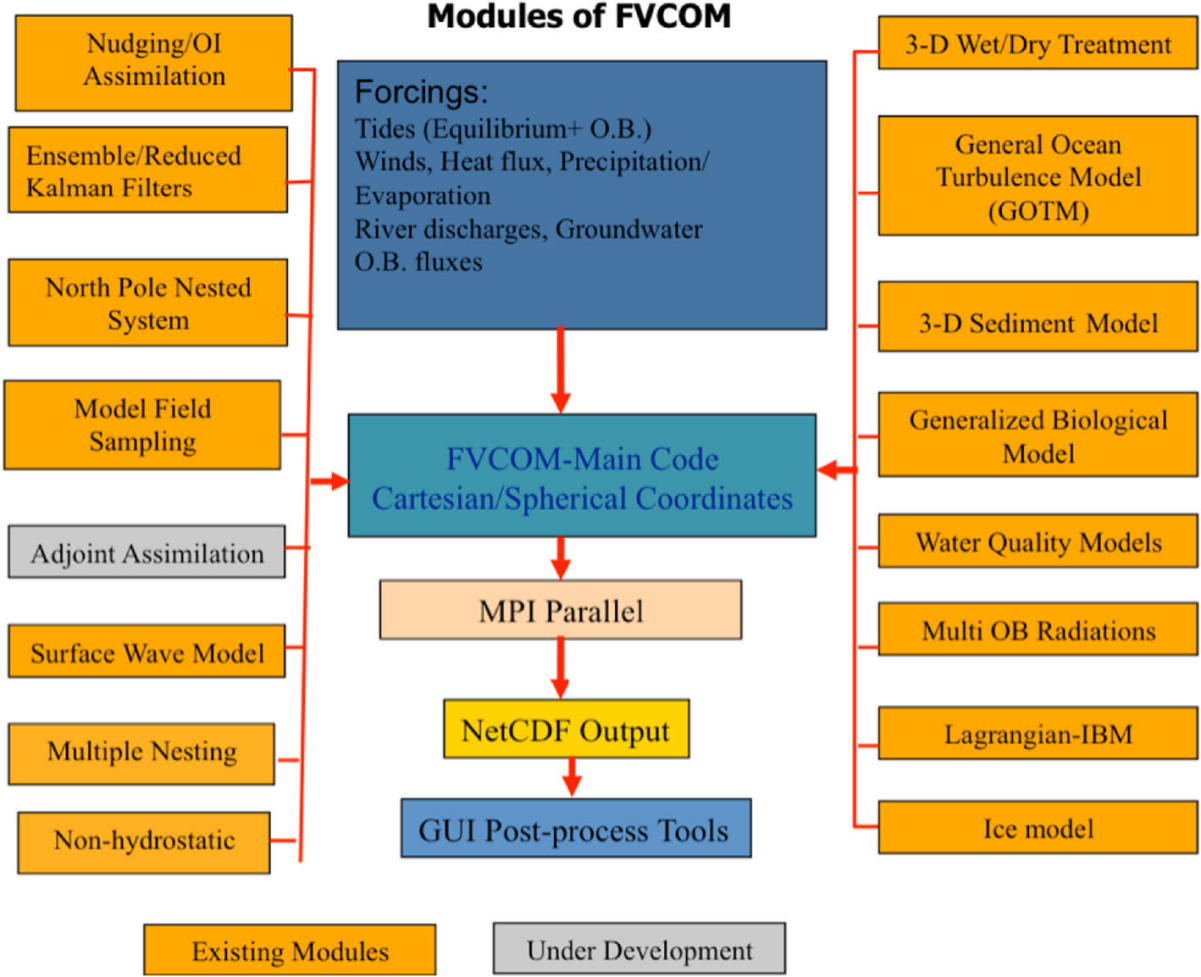
FVCOM program module structure (from [11])

FVCOM, an open-source 3D finite-volume code [11, 12], was used to represent the wind-driven circulation. FVCOM is designed to simulate time-dependent variation in water levels, currents, temperature, salinity, tracers, cohesive and non-cohesive sediments and waves in a variety of marine and freshwater systems. It is programmed in Fortran 90 and its modular structure allows a user to customise the model with just those routines required for a particular application (see Fig. 1). Although originally developed as an ocean model, FVCOM has been applied to a number of large lakes (e.g. [14, 48]), including a study of hydrodynamics and thermal structure in Lake Superior [70], and an examination of the influences of sediment transport on the ecosystem of Lake Michigan [12].

The basic equations are adapted to use a bottom following $\sigma$-coordinate system [6], based on transformation of the Cartesian coordinate *z* to gives a smooth representation of the bathymetry. The equations of mass, momentum and temperature conservation in $\sigma$-coordinates may be given as:

1 $$\frac{\partial \zeta }{\partial t} + \frac{\partial Du}{\partial x} + \frac{\partial Dv}{\partial y} + \frac{\partial \omega }{\partial \sigma } = 0$$

2 $$\frac{\partial uD}{\partial t} + \frac{\partial u^2 D}{\partial x} + \frac{\partial uv D}{\partial y} + \frac{\partial u\omega D}{\partial \sigma } - fvD = -\,gD\frac{\partial \zeta }{\partial x} - \frac{gD}{\rho _o}\left[ \frac{\partial }{\partial x}\left( D \int _\sigma ^0 \rho \,d\sigma ' \right) +\sigma \rho \frac{\partial D}{\partial x}\right] +\, \frac{1}{D}\frac{\partial }{\partial \sigma }\left( K_m \frac{\partial u}{\partial \sigma }\right) + DF_x$$

3 $$\frac{\partial vD}{\partial t} + \frac{\partial uv D}{\partial x} + \frac{\partial v^2 D}{\partial y} + \frac{\partial v\omega D}{\partial \sigma } - fuD = -gD\frac{\partial \zeta }{\partial y} - \frac{gD}{\rho _o}\left[ \frac{\partial }{\partial y}\left( D \int _\sigma ^0 \rho \,d\sigma ' \right) +\sigma \rho \frac{\partial D}{\partial y}\right] + \frac{1}{D}\frac{\partial }{\partial \sigma }\left( K_m \frac{\partial v}{\partial \sigma }\right) + DF_y$$

4 $$\frac{\partial TD}{\partial t} + \frac{\partial TuD}{\partial x} + \frac{\partial TvD}{\partial y} + \frac{\partial T \omega D}{\partial } = \frac{1}{D}\frac{\partial }{\partial \sigma }\left( K_h \frac{\partial T}{\partial \sigma }\right) + D\hat{H} + DF_T$$

5 $$\rho = \rho (T)$$

wherein the horizontal diffusion terms are defined as:

6 $$DF_x \,\approx\, \frac{\partial }{\partial x}\left[ 2A_m H \frac{\partial u}{\partial x} \right] + \frac{\partial }{\partial y}\left[ A_m H \left( \frac{\partial u}{\partial x} + \frac{\partial v}{\partial y} \right) \right]$$

7 $$DF_y \approx\, \frac{\partial }{\partial x}\left[ A_m H \left( \frac{\partial u}{\partial y} + \frac{\partial v}{\partial x} \right) \right] + \frac{\partial }{\partial y}\left[ 2A_m H \frac{\partial v}{\partial y} \right]$$

8 $$DF_T \approx\, \frac{\partial }{\partial x}\left( A_h H \frac{\partial T}{\partial x} \right) + \frac{\partial }{\partial y}\left( A_h H \frac{\partial T}{\partial y} \right)$$

where $D = H+\zeta$ is the water depth; *H* is the bottom topography; $\zeta$ is the surface elevation; *u*, *v* and $\omega$ are the velocity components in *x*, *y* and $\sigma$ directions respectively; *T* is the water temperature; $\rho$ is the density; *f* is the Coriolis parameter; $K_m$ is the vertical eddy viscosity coefficient; $K_h$ is the thermal vertical eddy diffusion coefficient; and $A_m$ and $A_h$ are the horizontal eddy and thermal diffusion coefficients, respectively. $K_m$ and $K_h$ are parameterized using the Mellor and Yamada [44] level 2.5 (MY2.5) turbulence closure scheme, as modified by Galperin et al. [21]. $A_m$ and $A_h$ are specified using the Smagorinsky parameterization method [61].

The boundary conditions for *u*, *v*, $\omega$ and *T* at the water surface ($\sigma = 0$) are defined as:

9 $$\left( \frac{\partial u}{\partial \sigma },\frac{\partial v}{\partial \sigma } \right)= \frac{D}{\rho _o K_m}(\tau _{sx}, \tau _{sy});\quad \omega = \frac{\hat{E} - \hat{P}}{\rho }$$

10 $$\frac{\partial T}{\partial \sigma }= \frac{D}{\rho c_p K_h} \left( Q_n (x,y,t) - SW(x,y,0,t)\right)$$

and at the bottom ($\sigma = -1$) as:

11 $$\left( \frac{\partial u}{\partial \sigma },\frac{\partial v}{\partial \sigma } \right)= \frac{D}{\rho _o K_m}(\tau _{bx}, \tau _{by});\quad \omega = \frac{Q_b}{\varOmega }$$

12 $$\frac{\partial T}{\partial \sigma }= \frac{A_h D tan \alpha }{K_h - A_h tan^{2} \alpha } \frac{\partial T}{\partial n}$$

where $\hat{P}$ and $\hat{E}$ are the precipitation and evaporation rates, respectively; $Q_n$ is the surface net heat flux; *SW*(*x*, *y*, 0, *t*) is the shortwave flux incident at the water surface; $Q_b$ is the groundwater volume flux at the bottom; $\varOmega$ is the area of the groundwater source; $c_p$ is the specific heat of water, $\alpha$ is the bottom slope; and *n* is a horizontal axis.

The shear stresses at the water surface are expressed as:

13 $${\tau _{sx} \atopwithdelims ()\tau _{sy}} = \rho _a C_{dw} {w_x \atopwithdelims ()w_y} {\sqrt{w_{x}^2 + w_{y}^2}}$$

where $w_x , w_y$ are the wind velocity components in *x* and *y* respectively, $\rho _a$ is the air density, and $C_{dw}$ is a drag coefficient specified within the model code and set equal to 0.0025.

The shear stresses at the bed due to currents are expressed as:

14 $${\tau _{bx} \atopwithdelims ()\tau _{by}} = \rho C_{d} {u \atopwithdelims ()v} \sqrt{u^2 + v^2}$$

where $C_d$ is a drag coefficient determined by matching a logarithmic bottom layer to the model at a height $z_{ab}$ above the bottom [11]; that is:

15 $$C_d = \max \left[ \frac{\kappa ^2}{\ln \left( \frac{z_{ab}}{z_o} \right) ^2} , 0.0025 \right]$$

where $\kappa = 0.4$ is the Von Karman constant and $z_o$ is the bottom roughness parameter.

### Case study description

**Fig. 2:**
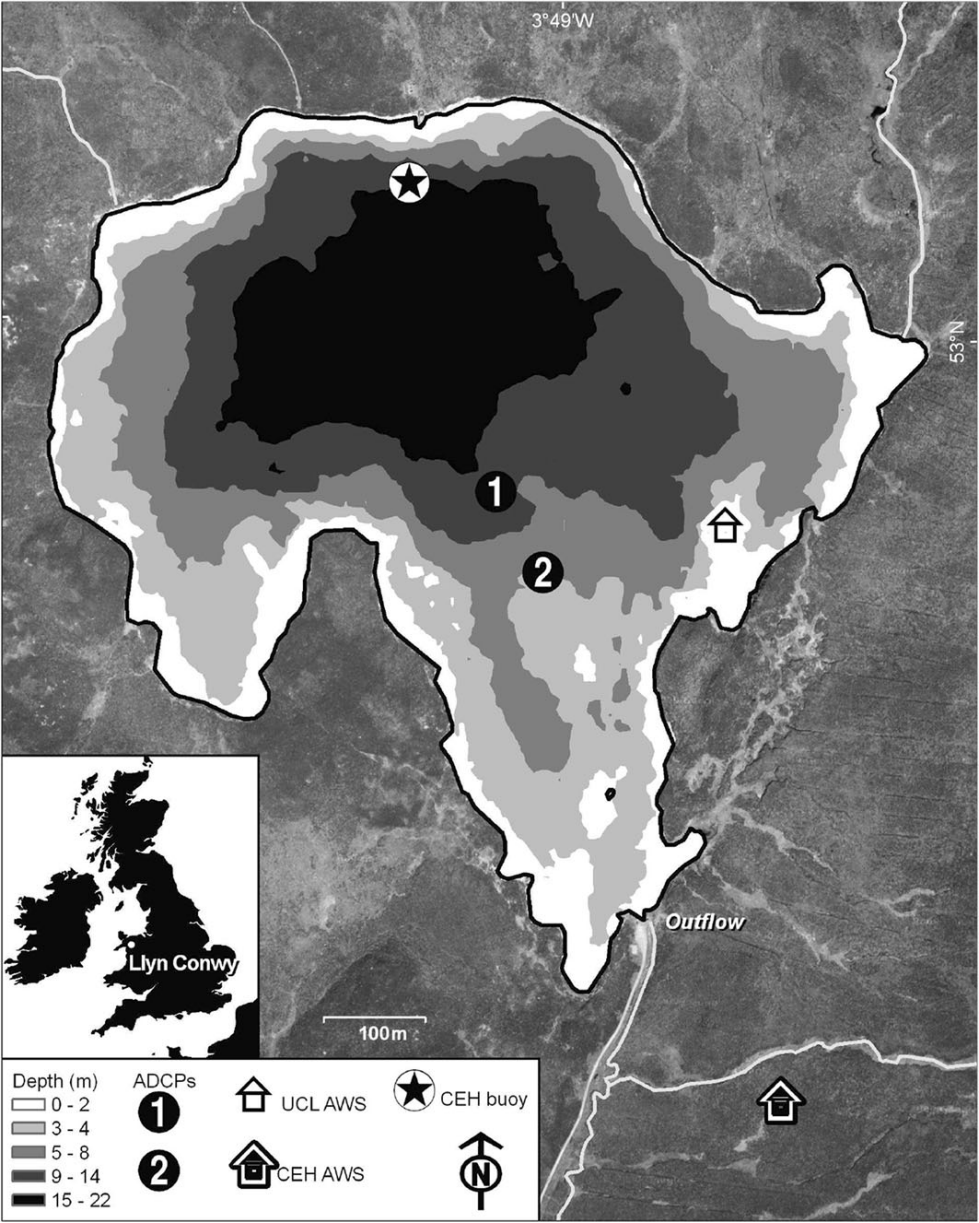
Llyn Conwy location, bathymetry (depth contours in m) and data acquisition locations. Automatic Weather Stations (AWS) recorded wind speed and direction, air temperature and pressure, and relative humidity. CEH data buoy includes an AWS and also a sensor string to measure vertical variation in water temperature. Acoustic Doppler Current Profilers (ADCPs) are upward-looking bottom deployments. See text for further information

Llyn Conwy is an upland lake 450 m above sea level in North Wales, UK (Fig. 2). The catchment is mainly blanket peat and its area is small (96 ha) relative to a lake area of 40 ha. Influxes are dominated by direct precipitation and seepage from the surrounding peat; tiny streams that enter the lake contribute a small fraction of the total water input and even after heavy rainfall are insignificant drivers of the water circulation. A single outflow drains into the River Conwy. Bathymetry is characterised by a central basin with shallower bays to the south and east. Mean and maximum water depth are approximately 7.7 and 22.0 m. Mean annual temperature and precipitation are approximately $10\,^{\circ }$C and 2300 mm [1]. The maximum catchment elevation is only 75 m above the lake surface and the lake is very exposed to the predominantly westerly to southwesterly winds. An automatic weather station (AWS) deployed in the catchment between 2006 and 2008 by the Centre for Ecology and Hydrology (CEH), Bangor, indicated a mean annual wind speed of about $10\,\text {m s}^{-1}$, with peak hourly wind speeds exceeding $30\,\text {m s}^{-1}$.

During winter, the lake is well mixed nearly 100% of the time because of the low incoming solar radiation and generally highwind speeds [46]. By the end of spring, the lake is stratified (> 70% of the time) under lower wind speeds and the rapid increase in shortwave solar radiation. Even in summer, persistent disruptions of the thermal stratification by periods of strong winds stir the water column. In autumn, the lake is weakly and intermittently stratified about 30% of the time as shortwave solar radiation declines and wind forcing becomes more energetic.

### Data acquisition

Two field campaigns were undertaken in July 2010 and April 2011 to acquire essential data on bathymetry, time series of meteorological forcing variables, and observations of surface water circulation and the vertical velocity structure. Hydrodynamic modelling is critically dependent on the quality of the bathymetry data [7]. Existing bathymetric data [52] were supplemented by additional surveys using a dGPS-equipped Raytheon single-beam sounding system (0.1 m resolution).

Meteorological data were obtained from a Davis Automatic Weather Station (AWS) equipped with sensors for wind speed ($\pm \, 3$% accuracy), direction ($\pm \, 7^\circ$ accuracy), air temperature (accuracy $\pm \, 1\, ^\circ$C), pressure (accuracy $\pm \, 1$ mB) and relative humidity (accuracy $\pm \, 3$%). The AWS was installed on a small island off the eastern shore of the lake, with the sensors approximately 2 m above the water level (Fig. 2). One of the main factors influencing the selection of Llyn Conwy as a case study was the availability of earlier data from an instrumented buoy deployed by CEH Bangor as part of a UK-wide upland lake monitoring programme. This included time series of water temperature from a sensor string in the deepest part of the lake (Fig. 2) that was equipped with 10 platinum resistance thermometers (accuracy $\pm \, 0.1\,^\circ$C) at 2 m intervals in the vertical. The CEH monitoring programme also included an AWS located on the buoy and another land-based AWS on the lake shoreline (see Fig. 2). A 20-year wind record for Capel Curig (UK Meteorological Office station 1171), 13 km northwest of Llyn Conwy, was used to undertake a more complete analysis of wind forcing variability. Data for this station were empirically corrected with reference to local Llyn Conwy wind data to take account of differences in location and altitude.

Vertical velocity profiles were obtained at two locations using a bottom-mounted (i.e. upward looking) RDI 1200KHz Workhorse Sentinel Acoustic Doppler Current Profiler (ADCP1-2 in Fig. 2). The ADCP was set to record 3D velocities at a vertical interval of 0.5 m, using ensembles of 150 pings to give a precision (standard deviation) of $\pm \, 0.057\,\text {m s}^{-1}$.

### Model setup and initial conditions

The unstructured triangular mesh comprised 1097 nodes and 2028 elements. The domain was divided into 12 $\sigma$ levels in the vertical. The small element size (mean edge length of approximately 13 m) meant that care had to be taken to ensure numerical stability when the wind forcing was very strong. Accordingly, a 1 s numerical timestep was used for all simulations in order to satisfy the Courant–Friedrich Levy (CFL) stability criterion. Similarly short numerical time steps have been used in other applications characterised by strong forcing conditions at the air-surface-water interface or at river inflow boundaries (e.g., [34]).

Calibration and validation were performed using datasets obtained in the April 2011 field campaign. Calibration utilized the ADCP1 deployment and validation the ADCP2 deployment. Additional simulations to further characterize the wind-driven circulation were undertaken using datasets from July 2010. The model was forced using meteorological data alone. Stream inflows were too small to measure (with inflow being dominated by slow seepage from peat outcrops around the entire shoreline) and the outflow was inactive during both field campaigns. Direct water inputs were thus neglected in the model runs reported here.

**Fig. 3:**
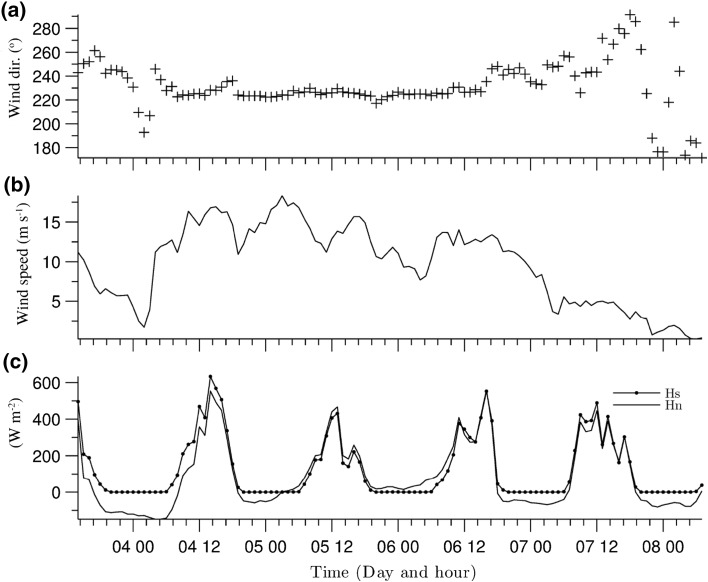
Hourly meteorological forcing from 03/04/2011 to 08/04/2011. **a** Wind direction; **b** wind speed measured 10 m above the ground; and **c** incoming solar radiation (Hs) and net heat flux (Hn), included here to provide a more complete indication of the meteorological forcing

For calibration purposes, *FVCOM* was forced by a 5-day sequence of meteorological data [wind direction, wind speed measured 10 m above the ground, incoming solar radiation (Hs) and net heat flux (Hn)] for April 2011 (Fig. 3). Winds are predominantly southwesterly (Fig. 3a), with speeds mainly between 12 and $16\text \,{ m s}^{-1}$ (Fig. 3b). The initial conditions were established by spinning up the model over two days using a constant wind speed ($11.2\text \,{ m s}^{-1}$) and direction (241.92$^\circ$), corresponding to the conditions at the start of the calibration dataset. A uniform distribution of the water temperature was imposed, equal to 4.71 $^\circ$C. This was based on analysing the thermal structure analysis (described in Morales-Marín [46]), which showed that in the month of April, the lake stays mixed more than 40% of the time, and during intermittent stratified periods, the thermocline is only weakly defined.

Full calibration of 3D hydrodynamic models is not always undertaken because of the high computational requirements (e.g. [4]), and use is often made of parameter values obtained from previous sensitivity analysis with simpler 1D and 2D models. Parameters such as bottom roughness ($z_0$) are established based on physical characteristics such as sediment grain size and density. For example in Clear Lake California, USA, where sediments are predominantly muddy, [54] set $z_0 = 0.02$ cm to implement a 2D model. In Lake Okeechobee, Florida, USA, a relative high value of $z_0 = 0.5$ cm was set to take account of abundant littoral vegetation [31]. Other studies (e.g. [22]) allow $z_0$ to vary with the local depth of based on the local depth, to account for the effects of wave motions in the hydrodynamic model. Despite an extensive literature on the roughness coefficient, its specification is often rather arbitrary.

Here, a calibration with respect to $z_0$ was carried out against the velocity profiles recorded using an ADCP at location 1 (ADCP1 in Fig. 2). $z_0$ was assumed to follow a uniform distribution between 0.001 and 0.06 m [29], with values sampled randomly using a Monte-Carlo approach. Model performance for 90 different values of $z_0$ was evaluated using the Nash–Sutcliffe Efficiency coefficient (NSE) [47], given by

16 $$NSE = 1-\, \frac{\sum _{i=1}^{n} (O_{i}-P_{i})^2}{\sum _{i=1}^{n} (O_{i}-\bar{O})^2}$$

where *O* are the observed and *P* are the predicted values. The subsequent analysis focuses on model output from three selected layers within the water column (surface, middle and bottom layers), which correspond to $\sigma$-levels of 0.1, 0.5 and 0.9 respectively.

## Results

### Model calibration

**Fig. 4:**
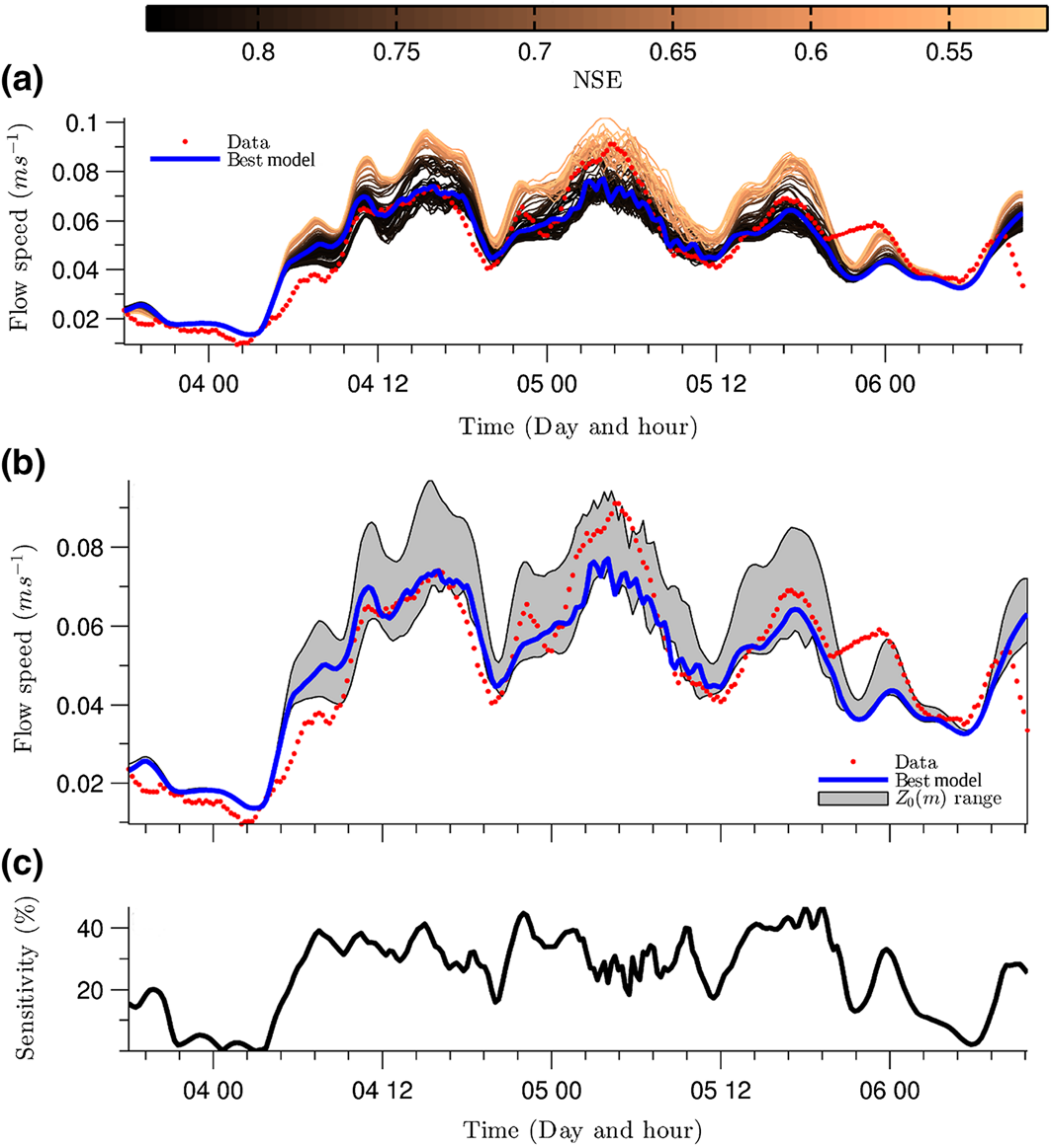
Calibration of $z_0$ based on comparison of simulated and observed depth-averaged velocities. **a** Comparison between hourly observed depth-averaged velocity time series at ADCP1 (see Fig. 2) and the simulated series for different values of $z_0$ (shaded lines). Best model simulation is plotted in blue; **b** depth-averaged observed (red dots) and simulated (blue line) velocity series. Shaded area represents envelope of model output for $z_0 = 0.001$ (upper line) to $z_0 = 0.06$ (lower line); and **c** Sensitivity Index (see text for explanation)

Calibration of $z_0$ was performed with respect to the NSE performance statistics computed from time series of hourly simulated and observed depth-averaged velocities at ADCP1 location (Fig. 4). The results of the calibration show a reasonable model performance (NSE > 0.5) for all the $z_0$ values. The calibration analysis showed that as the values of $z_0$ grow between 0.001 and 0.02 m, NSE increases rapidly, reaching its maximum value of 0.84 when $z_0 = 0.0227$ m. Then, NSE decreases only slightly to $\sim$ 0.80 for $z_0 > 0.0227$ m, indicating model insensitivity for that set of $z_0$ values.

Further analysis yields a correlation coefficient of $\sim$ 0.9 between the wind speed and the observed and best simulated depth-averaged flow velocity series. This shows that both the lake and the model are very sensitive, and respond quickly to, wind forcing changes.

Although the model performs well for the optimal $z_0$ values, it underestimates the velocity magnitudes by almost 25% at the velocity peak that occurred around 6 am on April 5th. In contrast, model over-estimation occurs around 6 am on April 4th and is nearly 30% at the end of the study period when the flow is relatively weak. The Sensitivity Index (e.g. [58]) shows also that the model is relatively sensitive ($> 30\%$) to changes of $z_0$ when flow velocities are greater than $0.05\ \text {m s}^{-1}$, and less sensitive ($< 20\%$) when flow velocities are lower (Fig. 4b). The behaviour of the Sensitivity Index suggests that the model performance would be improved if $z_0$ were $\le 0.005$ m for relatively high flow velocities and $\ge 0.01$ m for lower velocities.

### Model validation

**Fig. 5:**
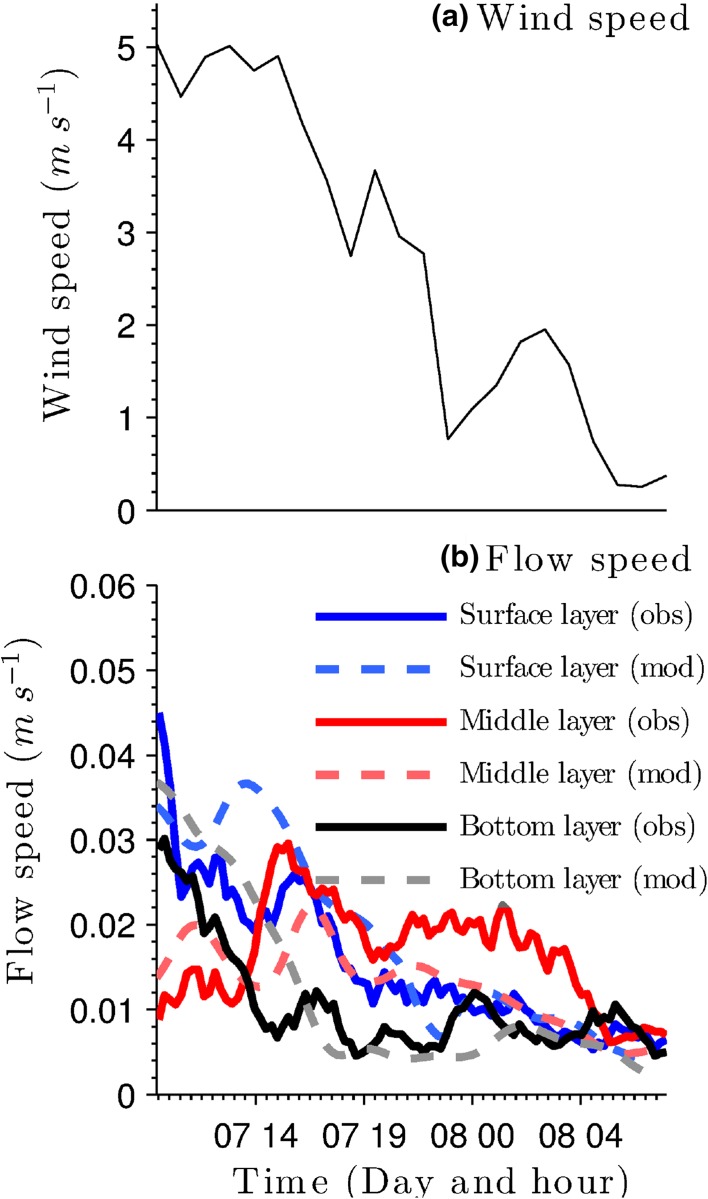
**a** Wind speed and **b** comparison between observed and simulated velocity magnitude at surface, middle and bottom $\sigma$ levels (corresponding to $\sigma = 0.1$, 0.5 and 0.9 respectively)

A separate validation was carried out against velocity profiles measured by the second ADCP deployment (ADCP2; Fig. 2). Observed and simulated flow series exhibit similar behaviour for three selected $\sigma$ levels with NSE $\sim$ 0.72 in all cases. The velocity series for the surface and middle layers (Fig. 5b) show a close correspondence with wind forcing (Fig. 5a). In contrast, the velocity series in the bottom layer shows insensitivity for wind speeds below 3 ms$^{-1}$.

**Fig. 6:**
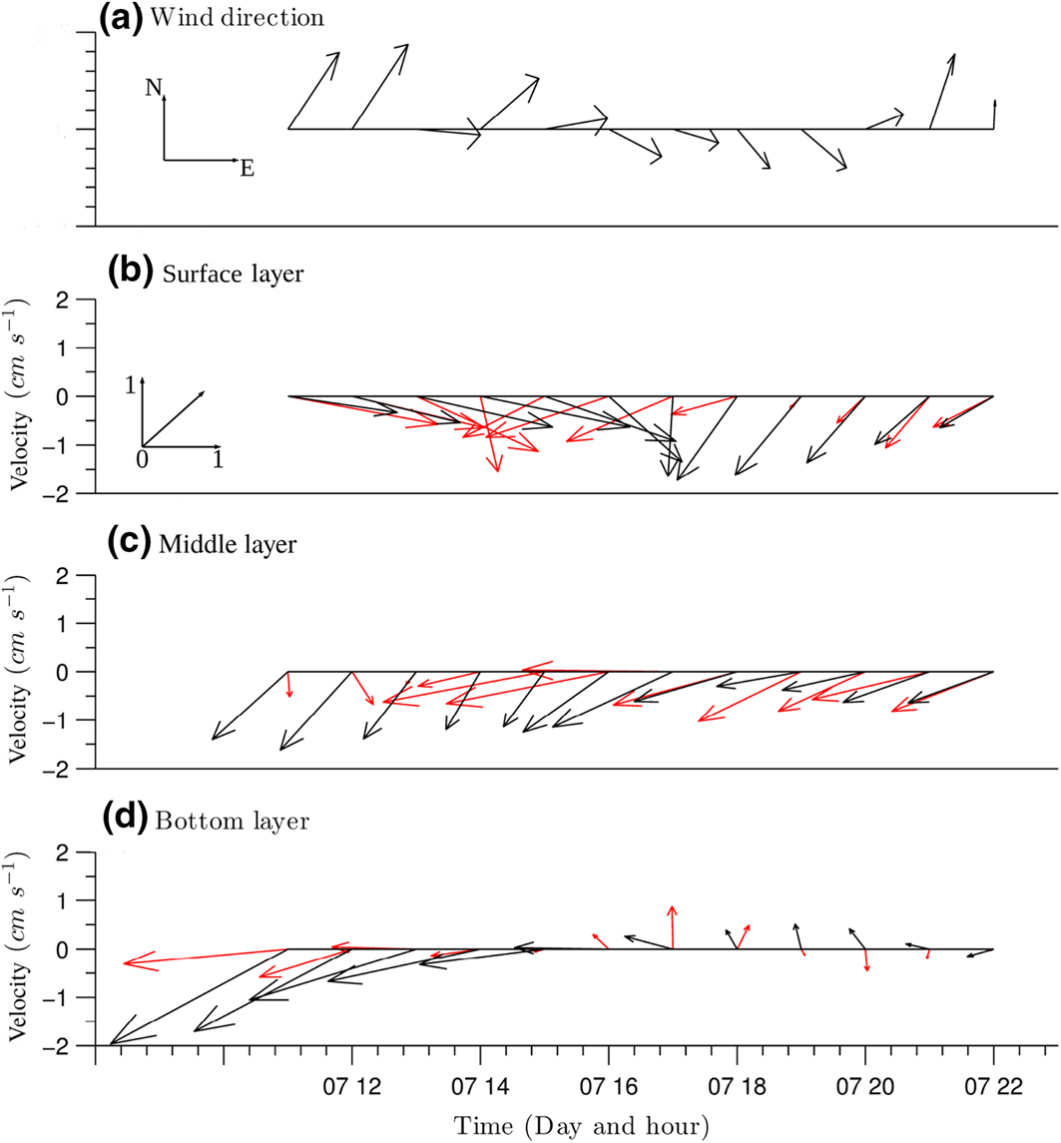
Comparison between observed (red) and simulated (black) current vectors at ADCP2 at top (**b**), middle (**c**) and bottom (**d**) $\sigma$ layers. Subplot **a** shows the time variation in the wind direction for the same period. A unit vector inset is include in subplot **b** to show the scale

A comparison between the *x* and *y* components of simulated and observed flow velocity reveals certain similarities (Fig. 6). In both the observed and simulated series, marked return flows, which oppose the prevailing wind direction, are observed. These occur during the whole period and are more intensive during the first 4 h when the wind speed is above 3 m s$^{-1}$. As wind speed decreases, the magnitude of the return flows also decreases and the flow velocity tends to zero.

Oscillatory motions are evident in both observed and modelled flow direction series (Fig. 6). In the first half of the period, flow vectors in the bottom and middle layers follow similar directions, especially in the return flows. In the surface layer, a small shift between the wind direction and flow direction occurs due to the bi-directional interaction of currents (Fig. 6a). In the second half of the calibration period, a return flow in the surface and middle layer is triggered by relaxation of the tilted water surface as wind speed decreases to $< 3\ \text {m s}^{-1}$. At the end of the second half of the calibration period, simulated and observed current vectors differ substantially in the bottom layer since the velocities here are relatively small ($<\,0.5\ \text {cm s}^{-1}$) and therefore more difficult to simulate in detail.

At the water surface, neither the simulated nor observed current vectors match the wind direction (Fig. 6b). Such behaviour is presumably caused because the wind forcing is relatively weak during the whole period and unable to drive the surface layer. This means that lake circulation and velocity field in general, are temporarily controlled by the topological and bottom stress moment respectively in the vorticity equation [37]. Studies of the mechanisms affecting circulation in Lake Michigan have similarly shown that, when the lake was forced with uniform wind field, the topographic effect was the dominating factor during unstratified periods [59]. On the other hand, a combination of internal waves and bottom topography was found to be the dominating factor in the set-up of meso-scale vorticity in Lake Stechlin, Germany [35]. A similar combination of circumstances might account for the discrepancies between wind and flow directions, in the second half of the Llyn Conwy dataset.

### Characterization of horizontal lake circulation

**Fig. 7:**
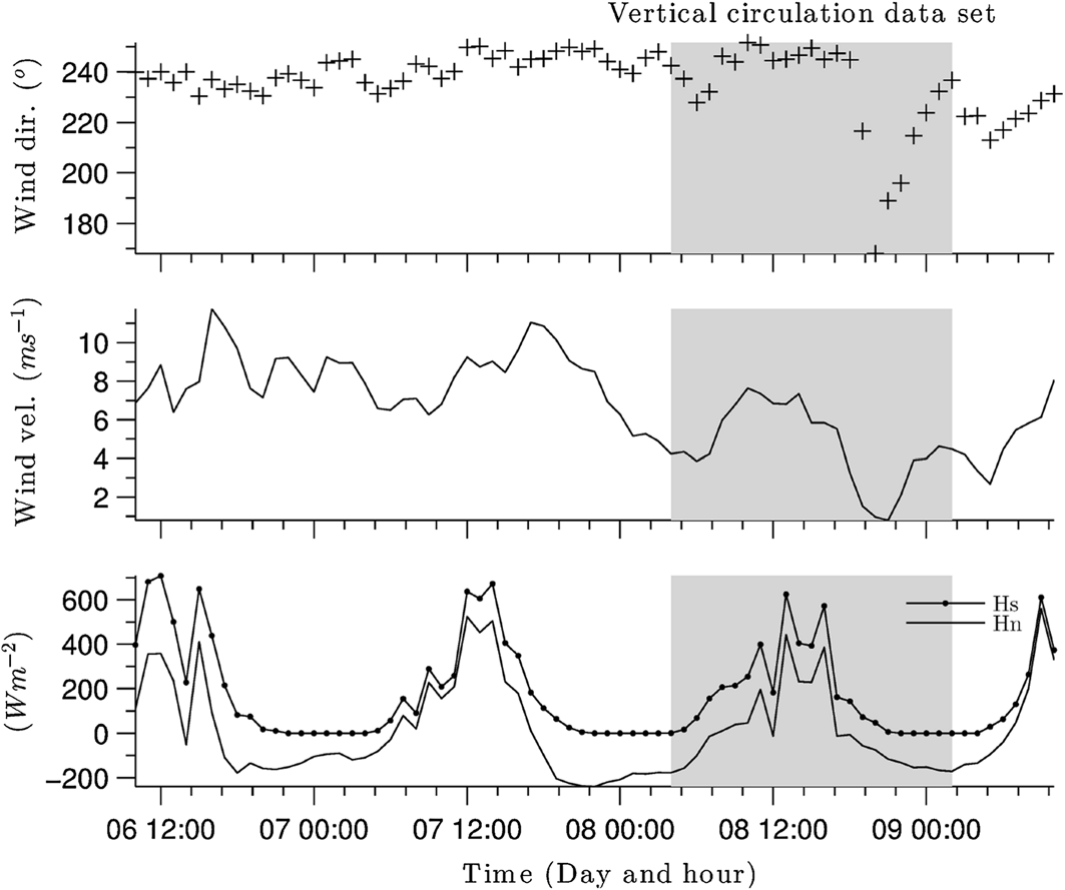
Meteorological forcing information from the first field campaign from 06/07/2010 to 09/07/2010. **a** Wind direction; **b** wind speed measured 10 m above the ground; and **c** incoming solar radiation (Hs) and net heat flux (Hn), included here to provide a more complete indication of the meteorological forcing. Shaded area indicates the data used to analyse vertical circulation (see Fig. 10)

In order to further investigate the nature of the wind-driven circulation, the calibrated model *FVCOM* was forced using meteorological data acquired during the first field campaign in July 2010 (Fig. 7). The initial conditions were established by spinning up *FVCOM* over four days using meteorological information corresponding to the first hour of the simulation period.

**Fig. 8:**
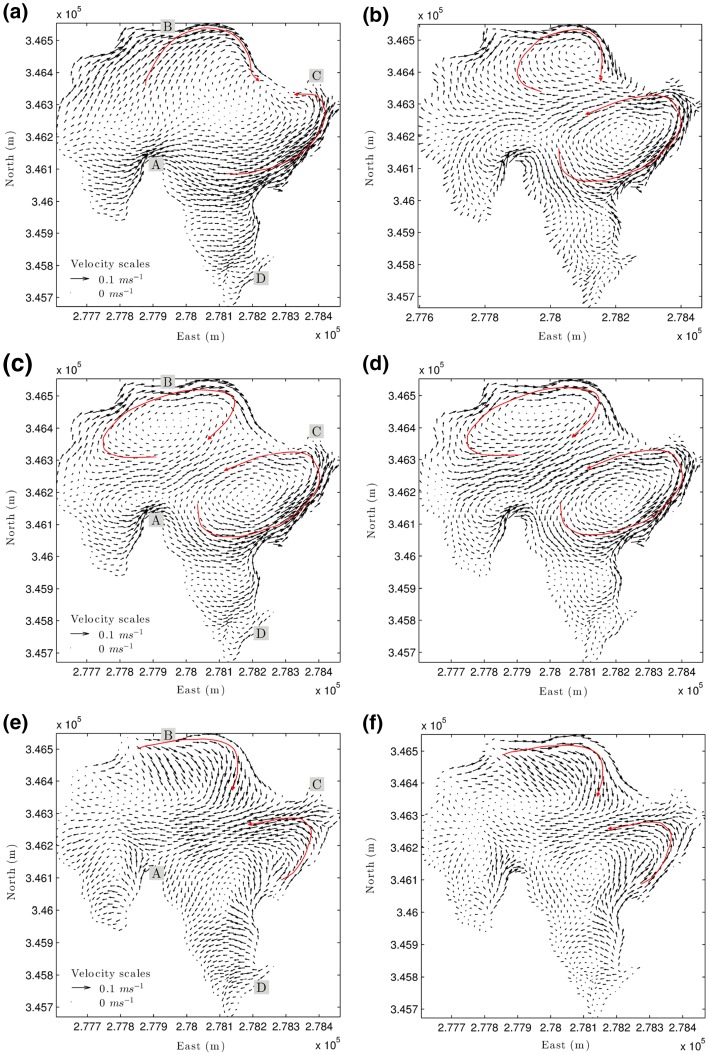
Time-averaged velocity fields simulated between 06-Jul-2010 and 09-Jul-2010 at **a** surface, **c** middle and **e** bottom layers and the field for EOF mode 1 at **b** surface, **d** middle and **f** bottom layers. Locations A to D in subplots **a**, **b**, **c** are referred to in the text. Main flow gyres are indicated in red

The horizontal circulation is best understood by time averaging the simulated velocity fields over the whole simulation period. Time-averaged velocity fields for the surface, middle and bottom layers are shown in Fig. 8. At the water surface, the circulation is essentially free of gyres (Fig. 8a). Instead, the flow within the surface layer increases along the dominant southwesterly wind direction, with regions of more rapid flow along the north shore (between locations B and C). At mid-depth, the time-average velocity field shows two different gyres: an anti-cyclonic (clockwise rotating) gyre in the northern part of the lake with a maximum velocity near B, and a cyclonic (counterclockwise) gyre formed in the east of the lake extending from A to C (Fig. 8c). Both gyres converge northwest of C and return toward the centre of the lake. The central return flow is completely developed at the bottom of the lake, where most of the bottom area is affected by currents that oppose the predominant wind direction (Fig. 8e).

Time-averaging of the velocity field provides a useful insight into the horizontal circulation. However, the persistence of the flow pattern may not be reliably inferred from simple averaging alone. A more robust approach is to use Empirical Orthogonal Function (EOF) analysis [18, 30] to remove the mean velocity field from each time step and then decompose it into principal circulation modes. EOF analysis (summarized in Table 1) shows that nearly 100% of the total variance (energy) is represented by the first five EOF modes, with mode 1 containing > 85 % of the variance. The concentration of the variance in a single mode implies that the circulation pattern does not actually vary greatly over time, which is not entirely unexpected given the persistent southwesterly wind forcing.

The EOF mode 1 at the water surface accounts for 89.7% of the total spatial variance (Fig. 8b). This mode shows two gyres: a small one toward the northeast and another toward the east. These gyres strengthen the mean circulation patterns in the north and east of the lake (see Fig. 8a). In contrast, weakening of the mean velocities in the south and west of the lake is due to the currents flowing in an opposite direction to the mean ones in the EOF mode 1. The EOF mode 1 for the middle layer, which accounts for 94.40% of the total spatial variance, shows two large gyres: one in the north and the other in the east of the lake (Fig. 8d). Both gyres strengthen the mean circulation in the middle layer especially near the centre of the lake. The current patterns shown by the EOF mode 1 for the bottom layer strengthen the mean circulation in the north and the flow from the east towards the centre of the lake (Fig. 8f).

**Table 1:** Variance ($\sigma ^2$) and cumulative variance ($\sum \sigma ^2$) of the first five EOF modes of the velocity field at three different layers in the water column

EOF mode	Surface layer		Middle layer		Bottom layer	
	Var. (%)	$\sum$ Var. (%)	Var. (%)	$\sum$ Var. (%)	Var. (%)	$\sum$ Var. (%)
1	89.70	89.70	94.40	94.40	85.10	85.10
2	4.50	94.20	2.10	96.50	7.00	92.10
3	2.45	96.65	1.45	97.95	4.35	96.45
4	1.70	98.35	1.00	98.95	1.95	98.40
5	1.45	99.80	0.85	99.80	1.35	99.75

**Fig. 9:**
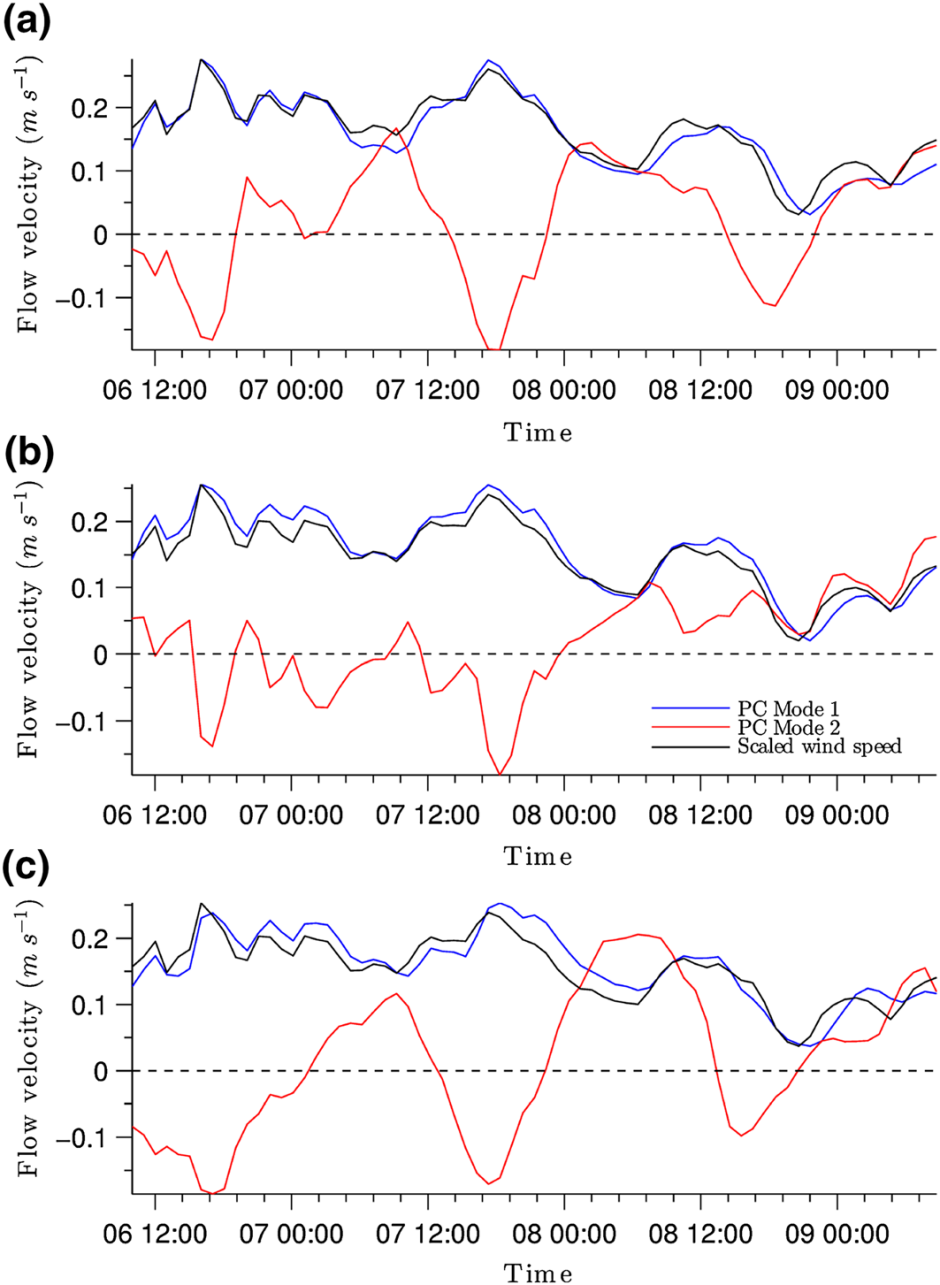
Principal components (PCs) of flow circulation patterns for EOF modes 1 and 2 at **a** surface, **b** middle and **c** bottom layers. Wind speed was scaled with respect to the magnitude of the PC mode 1 of flow velocity

The two-gyre pattern shown by EOF mode 1 at the surface and middle layers of the water column is mainly driven by the action of the predominantly southwesterly winds. The principal components (PCs) give the amplitudes through time of individual EOF modes. The PCs for EOF modes 1 and 2 both show a relationship with a scaled wind speed time (Fig. 9). The correlation coefficient is strongest for PC1 (about 0.95 for all layers). This confirms the strong dependency of lake circulation on wind forcing. Since PC is positive during the whole period, the two gyre patterns revealed by EOF mode 1 at the surface and middle layers can be considered to be the characteristic patterns in the lake (Fig. 8).

As already noted above, the EOF Mode 2 (PC2) partly counteracts EOF Mode 1 (PC1). At high wind speed events (e.g. around 18:00 on 06/07/2010), PC1 is strongly positive whereas PC2 shows negative values that counter large changes in wind forcing (Fig. 9). PC1 and PC2 are often out of phase during strong wind forcing changes, such that the combination of both modes creates a more complex circulation pattern.

### Vertical circulation

**Fig. 10:**
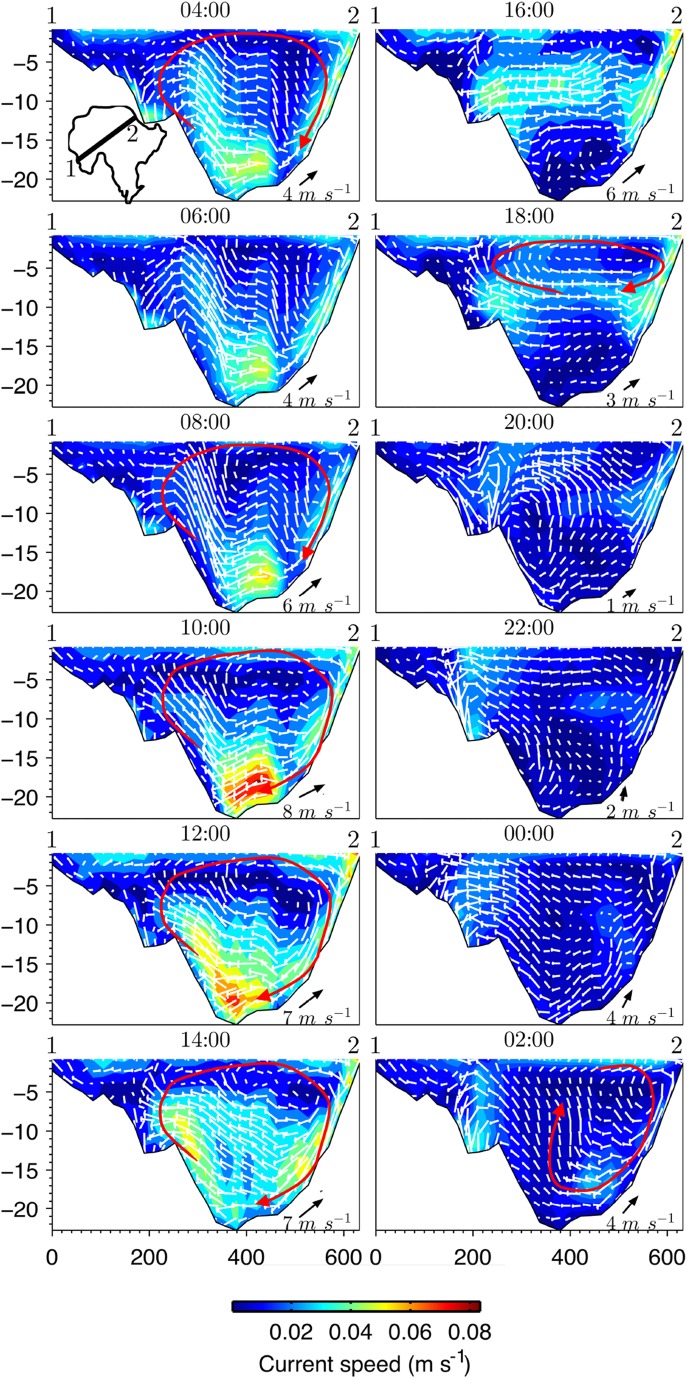
Transects of flow velocity from point 1 (upwind) to point 2 (downwind) (see inset at the top left) every 2 h starting from 06:00 07-Jul-2010 to 04:00 08-Jul-2010. Wind direction and speed are indicated at the lower-left corner of each panel. Velocity vectors are projected on the transect plane. Main flow circulation patterns are indicated in red

A 24-h period within the July 2010 dataset characterized by marked changes in wind speed and direction was selected for an additional analysis of the vertical circulation. The meteorological conditions in this period are indicated by the grey shaded area in Fig. 7. A sample transect along the predominant southwesterly wind track was chosen to analyse the time variation in vertical velocity structure. The analysis shows that the flow is generally accelerated by wind forcing from point 1 toward point 2, at which point a strong return flow commences along the downwind bed slope (Fig. 10). Observing the circulation between 08:00 and 12:00, during which time the wind speed rises from nearly 4 to 8 ms$^{-1}$, a large vertical circulation spanning the whole water column is initiated at $x = 200$ m (see Fig. 7). The maximum water velocities ($\sim \,0.08\ \text {m s}^{-1}$) occur at this time at the deepest part of the section. Given that the thermal stratification is intermittent and weak [46], the hypolimnion is weakly sheltered and the lake is susceptible to such intense vertical circulation events driven by brief periods of strong wind forcing. A noteworthy characteristic of the vertical circulation is that, in the shallowest regions (corresponding to the proximal end of the transect where $x < 200$ m), the velocities are always $< 0.01\ \text {m s}^{-1}$ because the return flow circulation does not extend this far and remains bounded by the deeper basin.

Another significant feature of the vertical circulation occurs when the wind speed drops below $4\ \text {m s}^{-1}$. Because the water column is not completely stirred, a more intense circulation develops in the upper layers and a weaker one in the lower ones. This is shown by the cross-sectional plots at times 16:00 and 18:00 in Fig. 10. The upper circulation, which extends from 0 to −8 m below the water surface, leads to the formation of a mixed layer that is bounded by the deeper parts of the section. The deeper and weaker circulation, which extends from −8 to −21 m depth and rotates counter-clockwise, may partly reflect the remnants of past circulation events across the whole water column that were driven by strong wind forcing events. This weaker circulation tends to dissipate as it loses energy by friction at the bed and against the circulation enclosed within the upper mixed layer.

The double vertical circulation is disrupted by significant changes in wind direction. Looking at the wind forcing series between 18:00 and 20:00, the wind direction shifts by 70$^\circ$ from a southwesterly to a southeasterly direction. Around this time, the double circulation pattern is substantially disrupted, leading to the formation of a unique anti-clockwise circulation and a chaotic flow field at the interface between the shallow and deep regions between $200\,<\,x\,<\,300$ m (see cross-sectional plot at 20:00 in Fig. 10). This chaotic flow field is formed when the quiescent current flowing from point 1 meets the energetic new vertical circulation, yielding a turbulent regime at the interface. This chaotic interface decays and the currents tend to recover their characteristic vertical circulation as the wind shifts back to a more typical southwesterly direction.

## Discussion

### Model calibration and validation

An *FVCOM* hydrodynamic model was successfully calibrated by optimizing the bottom roughness coefficient ($z_0$). This yielded an optimum $z_0 = 0.0227$ m. The relatively high value of $z_0$ ca be explained by the fact that the lake bed is highly irregular and the bottom sediment coverage and composition quite heterogeneous, as shown by grab sampling and coring carried out by Morales-Marín [46]. Similarly, high values of $z_0$ were also obtained by Gross et al. [22] in their calibration of a 3D hydrodynamic model of San Francisco Bay, and were attributed to the presence of a rough bed in shallower areas, which served to dampen wind-wave propagation.

Evaluation of overall model performance against its ability to reproduce time-series current velocities resulted in a value of $\text {NSE} = 0.84$, which is very good in terms of the criteria for the NSE statistic presented by Henriksen et al. [25]. Despite the generally good model performance, the model underestimates the current velocities by nearly 25% in the middle and near the end of the calibration period, when the velocities reach their peak values. These discrepancies might be partly due to the uncertainties in the meteorological forcing and the velocity profile measurements (which are quite challenging for such slow flows). The assumption of a homogenous and time-invariant bottom roughness may also be a factor. A further sensitivity analysis showed that the model underestimation at flow speeds $> 0.05\ \text {m s}^{-1}$ can be overcome if $z_0$ is $\sim$ 0.005 m. Similar dependence of the roughness parameter on flow velocity magnitude has been noted in the calibration of 1D/2D hydrodynamic models [27, 51].

### Lake circulation patterns

The gross circulation simulated using the validated model exhibits two distinct gyres that extend throughout the upper half of the water-depth layer. Analysis of the persistence of this typical two-gyre circulation pattern using Empirical Orthogonal Functions (EOFs) shows that EOF mode 1 accounts for more than 85% of the variance of the circulation. This implies that the circulation pattern is driven mainly by the predominant south-westerly wind forcing. The anti-cyclonic gyre covers the centre-north of the lake and flows intensively along the north shore. The cyclonic gyre located towards the east of the lake flows more rapidly along the east shore and returns toward the centre with great intensity. There, it merges with the anti-cyclonic gyre and flows smoothly toward the west of the lake. This western flow is completely developed at the lower water-depth layers, where return currents from the north to the centre of the lake are also apparent. The circulation patterns are therefore mainly produced by the interaction of wind forcing, the water body and the bathymetry.

Other studies of vorticity in lakes have found that bottom stresses and advection-diffusion act as sinks of vorticity, whereas wind stress gradients, internal pressure gradients (including temperature gradients) and the Coriolis force can be regarded as sources [37, 55, 59, 65]. The balance of these sources and sinks, as determined by weather conditions, lake catchment topography and the bathymetry, define the vorticity or circulation pattern in the lake. For example, Schwab and Beletsky [59] found that, in Lake Michigan, a cyclonic circulation occur in winter (unstratified period) caused by a wind stress curl. In summer, the cyclonicity is maintained but with slight variations due to non-linearities introduced by the baroclinicity. In Lakes Ontario and Erie, Beletsky et al. [4] observed two cyclonic gyres during winter, which were triggered by a non-uniform wind field. In Lake Erie, which is shallower than Ontario, anticyclonic circulation was observed due to an anticyclonic wind stress curl induced by a meso-scale high pressure gradient over the lake. Studies of Lake Kinneret and Lake Constance [60], and Lake Geneva [39], show that their circulation patterns are also driven by wind stress curls. In smaller lakes such Lake Stechlin, Germany, the interaction between basin topography and wind-induced seiches controls the formation of mesoscale vortices [35].

Modelling of wind-driven circulation is often based on the assumption of a spatially uniform wind stress at the water surface. Even in small lakes, however, it has been demonstrated that the circulation can be strongly affected by topographic sheltering. A study by Podsetchine and Schernewski [53] of Lake Belau, a small $1.1\ \text {km}^2$ lake in Northern Germany, compared the circulation patterns generated by uniform and non-uniform wind stress fields interpolated using data from two meteorological stations located on the shore and at the lake centre. The interpolation assumed a zero stress along the upwind shores and a downwind linear growth of the wind stress along the fetch. The results indicated that local topography and sheltering from surrounding forest (see also [42, 43]) creates a non-uniform wind field that drives a single gyre circulation in the lake. In contrast, imposition of a spatially uniform wind field yields a two-gyre circulation. Topographic sheltering is probably less important at Llyn Conwy, given that the maximum elevation within the tree-less catchment is only 75 m above the lake surface. However, other factors such as lake shape [36], surface roughness heterogeneity and differential wind acceleration along the fetch [67] can also create inhomogeneous wind fields.

### Effect of spatially varied wind forcing

In the light of the preceding remarks on the potential effect of topographic sheltering on the wind field, a further model experiment was conducted to investigate the differences between the circulation patterns generated by uniform and non-uniform wind stress field at Llyn Conwy. An initial scenario assumed a uniform wind stress field driven by $W_s = 12\ \text{m s}^{-1}$ and $W_d = 210^\circ$. A second scenario considered a non-uniform wind stress field generated by allowing wind speed to grow linearly along the southeasterly wind track from an initial value imposed at the western (upwind) shore. The two wind field scenarios were then used to force *FVCOM* in steady-state mode.

**Fig. 11:**
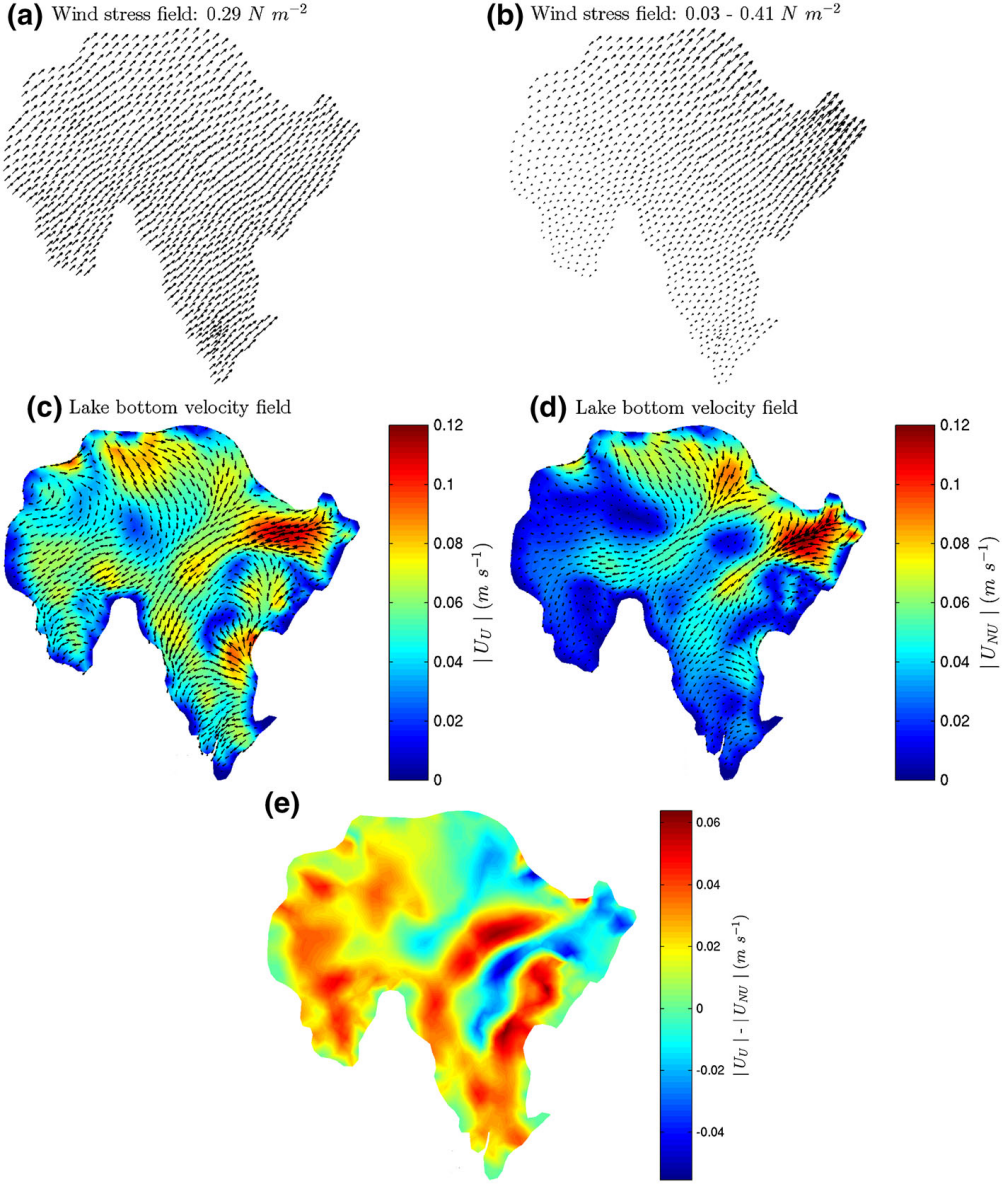
Comparison between bottom current velocities generated by **a** uniform and **b** non-uniform wind stress fields for $W_s = 12\ \text {m s}^{-1}$ and $W_d = 225^\circ$; **c** bottom velocity field for uniform wind stress distribution; **d** bottom velocity field for non-uniform wind stress distribution; **e** difference between velocity magnitude for uniform and non-uniform wind stress fields

As shown in Fig. 11, results for the uniform wind field scenario show strong bottom currents over approximately the whole bottom lake, whereas for the non-uniform wind field scenario, significant bottom currents are only generated in the east and northeast parts of the lake. Noticeable differences between the two forcing scenarios occur in shallow, short-fetch, areas in the west and south (Fig. 11e). Circulation patterns are also different in that only one return flow originates from the northeast under the uniform wind scenario, compared to two under the non-uniform scenario. Although subtle, these differences might have important implications for the pattern of sediment accumulation. The northern and western flow patterns given in both scenarios are consistent with areas that appear to be free of lake deposits in the north [46]. However, toward the shallower western and southern sectors, the less energetic circulation generated by a non-uniform wind field is more consistent with the sediment accumulation that this observed in these areas [46]. More detailed meteorological data are needed to full investigate these effects (e.g. [49, 55]). Such data collection should ideally become routine in physical limnological studies, even for small lakes such as Llyn Conwy.

## Conclusions

A community numerical ocean model, *FVCOM*, has been used as a platform to extend the understanding of wind-driven circulation in small upland lakes. A 3D model for a case study lake (Llyn Conwy, Wales, UK) is calibrated against measured velocity profiles via adjustment of the bottom roughness coefficient. Validation against a separate set of measured velocity profiles confirms the ability of the calibrated model to resolve key aspects of the flow field. Sensitivity analysis shows that the flow field within the lake responds rapidly to changes in the wind forcing. Analysis of the gross circulation using Empirical Orthogonal Functions reveals a persistent two-gyre circulation pattern in the upper half layer of the water column driven by the interaction of a predominant south-westerly wind and the irregular bathymetry. At the bottom, the flow is characterised by locally strong currents and analysis of vertical circulation over short time scales shows strong currents in the deepest parts of the lake basin and the responsiveness of the water column to changes in wind speed and direction. Even in small lakes, the assumption of uniform wind stress across the water surface is not always justified and topographic sheltering or other catchment roughness effects give rise to heterogeneity in the wind field. An initial analysis at Llyn Conwy shows that subtle differences in circulation emerge if the wind stress is allowed to vary across the lake.

Palaeoenvironmental, ecological and biogeochemical studies of upland lakes are often carried out in the absence of a sound understanding of the physical limnology. Energetic wind forcing in upland areas can drive an energetic lake circulation that has important implications for mixing and sediment dynamics. Numerical modelling of wind-driven circulation can provide valuable support for these studies and should be more widely used. Further work is needed to generalize the case study results to a wider range of upland lakes of contrasting size, geometry and exposure to wind. More detailed studies of wind forcing heterogeneity, including topographic sheltering and its effects on lake circulation and thermal stratification, are also needed.
